# Classification of Alzheimer’s disease progression based on sMRI using gray matter volume and lateralization index

**DOI:** 10.1371/journal.pone.0262722

**Published:** 2022-03-30

**Authors:** Qian Zhang, XiaoLi Yang, ZhongKui Sun

**Affiliations:** 1 College of Mathematics and Statistics, Shaanxi Normal University, Xi’an, 710119, PR China; 2 Department of Applied Mathematics, Northwestern Polytechnical University, Xi’an, 710129, PR China; University of North Carolina at Chapel Hill, UNITED STATES

## Abstract

Note that identifying Mild Cognitive Impairment (MCI) is crucial to early detection and diagnosis of Alzheimer’s disease (AD). This work explores how classification features and experimental algorithms influence classification performances on the ADNI database. Based on structural Magnetic Resonance Images (sMRI), two features including gray matter (GM) volume and lateralization index (LI) are firstly extracted through hypothesis testing. Afterward, several classifier algorithms including Random Forest (RF), Decision Tree (DT), K-Nearest Neighbor(KNN) and Support Vector Machine (SVM) with RBF kernel, Linear kernel or Polynomial kernel are established to realize binary classification among Normal Control (NC), Early Mild Cognitive Impairment (EMCI), Late Mild Cognitive Impairment (LMCI) and AD groups. The main experimental results are as follows. (1) The classification performance in the feature of LI is poor compared with those in the feature of GM volume or the combined feature of LI and GM volume, i.e., the classification accuracies in the feature of LI are relatively low and unstable for most classifier models and subject groups. (2) Comparing with the classification performances in the feature of GM volume and the combined feature of LI and GM volume, the classification accuracy of NC group versus AD group is relatively stable for different classifier models, moreover, the accuracy of AD group versus NC group is almost the highest, with the most classification accuracy of 98.0909%. (3) For different subject groups, the SVM classifier algorithm with Polynomial kernel and the KNN classifier algorithm show relatively stable and high classification accuracy, while DT classifier algorithm shows relatively unstable and lower classification accuracy. (4) Except the groups of EMCI versus LMCI and NC versus EMCI, the classification accuracies are significantly enhanced by emerging the LI into the original feature of GM volume, with the maximum accuracy increase of 5.6364%. These results indicate that various factors of subject data, feature types and experimental algorithms influence classification performances remarkably, especially the newly introduced feature of LI into the feature of GM volume is helpful to improve classification results in some certain extent.

## Introduction

Alzheimer’s disease (AD) is an irreversible progressive neurodegenerative disease in the brain. Mild Cognitive Impairment (MCI), as a prodromal stage of AD, is the most challenging group, which can be divided into Early Mild Cognitive Impairment (EMCI) and Late Mild Cognitive Impairment (LMCI). It is well known that MCI has a high risk of developing into AD, thus identifying MCI is key to early detection and diagnosis of AD [1].

In recent years, there has been great effort to identify pathological markers of AD and MCI so as to better computer-aided diagnosis or prognosis of them. With the rapid development of neuroimaging technology, various kinds of neuroimaging modalities including magnetic resonance imaging (MRI), positron emission tomography (PET), electroencephalogram (EEG) and cerebrospinal fluid (CSF) have been widely applied to the auxiliary prediction and diagnosis of AD [2–5]. Particularly, structural MRI (sMRI), for its non-invasive and convenient to provide visual brain atrophy information of macroscopic tissue and structure, has become more and more popular in clinical diagnosis of AD [6–8]. Some researchers have devoted to distinguishing AD and MCI with sMRI data using different features and achieved some good classification results. For example, based on the structural image data of the subjects, Beheshti et al. have made the classification accuracy of AD versus NC reach 84.07% by extracting the feature of the gray matter similarity matrix. Then the Mini-mental State Examination (MMSE) scale has been further considered, and the classification of patients with AD versus NC has reached the accuracy of 97.01% [9]. Tang et al. have selected shape differences and heterosexual fraction values of hippocampus and amygdala as features, and achieved the accuracy of 96.4% in AD versus NC classification experiments with SVM classifier [10]. By selecting Automated Anatomical Labeling (AAL) template as Region of Interest (ROI), previous works have taken functional connectivity as feature and achieved the classification accuracy of 82% and 91% for AD versus non-AD and NC versus MCI subjects, respectively [11,12]. Visser et al. have found that patients with MCI have medial temporal atrophy and memory impairment, and the feature of memory impairment is a better predictor of dementia than that of medial temporal lobe, moreover, the combination of them can improve the prediction accuracy of AD [13].

As well known that loss of nerve cells and synapses is common in MCI and AD patients, which results in the phenomenon of brain atrophy. The degree of brain atrophy can be effectively measured by gray matter (GM) volume. Some works in the literature have validated that gray matter in global and local brain regions is lost for MCI and AD subjects [14–16]. Through the follow-up of NC or MCI subjects and the measurement of the volume of gray matter in hippocampus, amygdala or temporal lobe, previous works have reported that the elderly will have physiological brain atrophy with age [17,18]. Butto et al. have found that the temporal lobe gray matter is decreased in AD patients by voxel-based morphometric (VBM) method [19]. Thus, gray matter volume has been employed as a kind of anatomical feature to effectively assist early prediction of AD. By classifying the gray matter volume of some brain structures, Schmitter et al. have improved the classification accuracy of AD versus MCI group and early AD versus late AD group, respectively [20]. By combing the gray matter volume with average intensity and CSF, Suk et al. have confirmed the classification accuracy of AD is greatly improved based on MRI and PET [21].

On the other hand, there exist left and right asymmetry in brain structure and function which is termed as the phenomenon of lateralization. Ratnarajah et al. have studied the asymmetry of brain structural connection in normal newborns [22]. Iturria-Medina et al. have explored the asymmetry of hemispheric structures between human and nonhuman primates [23]. By introducing the lateralization index to quantify the asymmetry, Kim et al. have found the cortical asymmetry between NC, MCI and AD patients [24]. Though the asymmetry is of significance in brain structure and function, this characteristic of asymmetry has rarely been attempted to recognize AD, especially combining the lateralization index with some anatomical features to assist prediction and early diagnosis of AD. On the same time, previous works have indicated that the classification accuracy of AD and MCI is usually influenced by various factors including subject data, feature type and experimental algorithms [25,26]. Therefore, in this work we devote to selecting the lateralization index and the GM volume as features, and then establishing several classifier models including Random Forest (RF), Decision Tree (DT), K-Nearest Neighbor(KNN) and Support vector machine (SVM) with RBF kernel, Linear kernel or Polynomial kernel, so as to realize binary classification prediction among NC, EMCI, LMCI and AD subjects from the Alzheimer’s Disease Neuroimaging Initiative (ADNI) database. The main purpose of this work is to explore how the classification features and classifier models affect the classification accuracy.

The structure of this work is as follows. Section 2 presents preparatory work and classification algorithm used in this work. The classification performances are illustrated in Section 3 in detail. Finally, conclusion and discussion are address in Section 4.

## Preparatory work and classification algorithm

In this section, some preparatory works for the classification experiment are firstly present, then the detailed experimental algorithm is illustrated.

### Data acquisition

Experimental data employed in this work are obtained from the ADNI database (ADNI, http://adni.loni.usc.edu/). The primary purpose of ADNI is to collect, validate and utilize data, including MRI and PET images, genetics, cognitive tests, CSF and blood biomarkers, so as to define the progression of AD [2,5,27]. ADNI results from combined efforts of numerous co-investigators from an extensive range of academic institutions and private corporations. Subjects recruiting in ADNI include Alzheimer’s disease patients, mild cognitive impairment subjects and elderly controls.

In the present work, the data sample contains 203 structural MRI images from 94 subjects ranged from 55 to 90 years old from the ADNI dataset. For each subject multiple images are collected at different time points. As indicated by the ADNI dataset, the cognitive function outcomes of MMSE (Mini-Mental State Examination) and CDR (Clinical Dementia Rating) for the same subjects vary in different ages. Previous works reported that in the follow-up of NC or MCI subjects the elderly will have physiological brain atrophy with age by measuring the volume of gray matter in hippocampus, amygdala or temporal lobe [17,18]. These findings indicates that the MRI scanning status of the same subject may change over time. Among these structural images, 67 images are for 27 NC subjects (12 males and 15 females), 58 images are for 27 EMCI subjects (18 males and 9 females), 39 images are for 18 LMCI subjects (10 males and 8 females) and 39 images are for 22 AD subjects (12 males and 10 females). The three dimensional T1-weighted anatomical magnetization prepared rapid gradient echo (MPRAGE) imaging parameters are as follows: the three dimension of sMRI images is 256×256×170, the flip angle is 9°, the slice thickness is1.2mm, the TR/TE is 6.8/3.1 and the voxel size is 1mm×1mm×1.2mm.

### Image pre-processing

Pre-processing of MRI samples is performed in MATLAB 2016b environment by Computational Anatomy Toolbox version 12 (CAT12, http://dbm.neuro.uni-jena.de/cat) [28]. CAT 12 is a popular neuroimaging analysis software that can realize the Voxel-based Morphometric (VBM) pipeline, which runs in the Statistical Parametric Mapping toolbox version12 (SPM12). It is commonly applied to investigate the distribution of GM and calculate the volume of GM. The detailed processing steps include format conversion, skull removal, tissue segmentation, dartel registration and normalization. Then based on the AAL atlas the GM volume of 90 brain anatomical are acquired.

### Lateralization index

As stated in the Introduction, the phenomenon of lateralization exists in brain for its left and right asymmetry of structure and function. The asymmetry is quantified by lateralization index. The Lateralization Index (LI) for gray matter volume is defined as follows [24]

$$\text{LI}=\frac{L-R}{L+R}\times 100$$

Where L and R represent the values of gray matter volume of the corresponding brain regions in the left and right hemispheres, respectively. This LI can quantitatively measure the difference of GM volume between the left and right hemispheres of the brain. A positive LI means that the GM volume for the left hemisphere is more prominent than the right hemisphere. The brain region with positive LI is called the left asymmetric region, otherwise referred to as the right asymmetric region.

### Feature extraction

It’s necessary to extract feature for image classification. In this work, two hypothesis testing methods, i.e., one way ANOVA and the independent sample non-parametric test of Kruskal-Wallis, are employed to perform features extraction using 203 structural MRI scans [29]. For the samples of GM volume and LI in every brain region for four groups of NC, LMCI, EMCI and AD subjects, the condition of normality and variance homogeneity are firstly examined. If this condition is met, the ANOVA as well as the LSD-t multiple comparisons are carried out for the samples within one brain region so as to check whether there is significant difference between the four groups. Otherwise, the Kruskal-Wallis test is conducted. The threshold p value is set to 0.05 in the above hypothesis test. On the basis of IBM SPSS Statistics 25, the features including 64 indicators for the GM volume and 15 indicators for the LI are obtained.

### Classification algorithm

**Fig 1:**
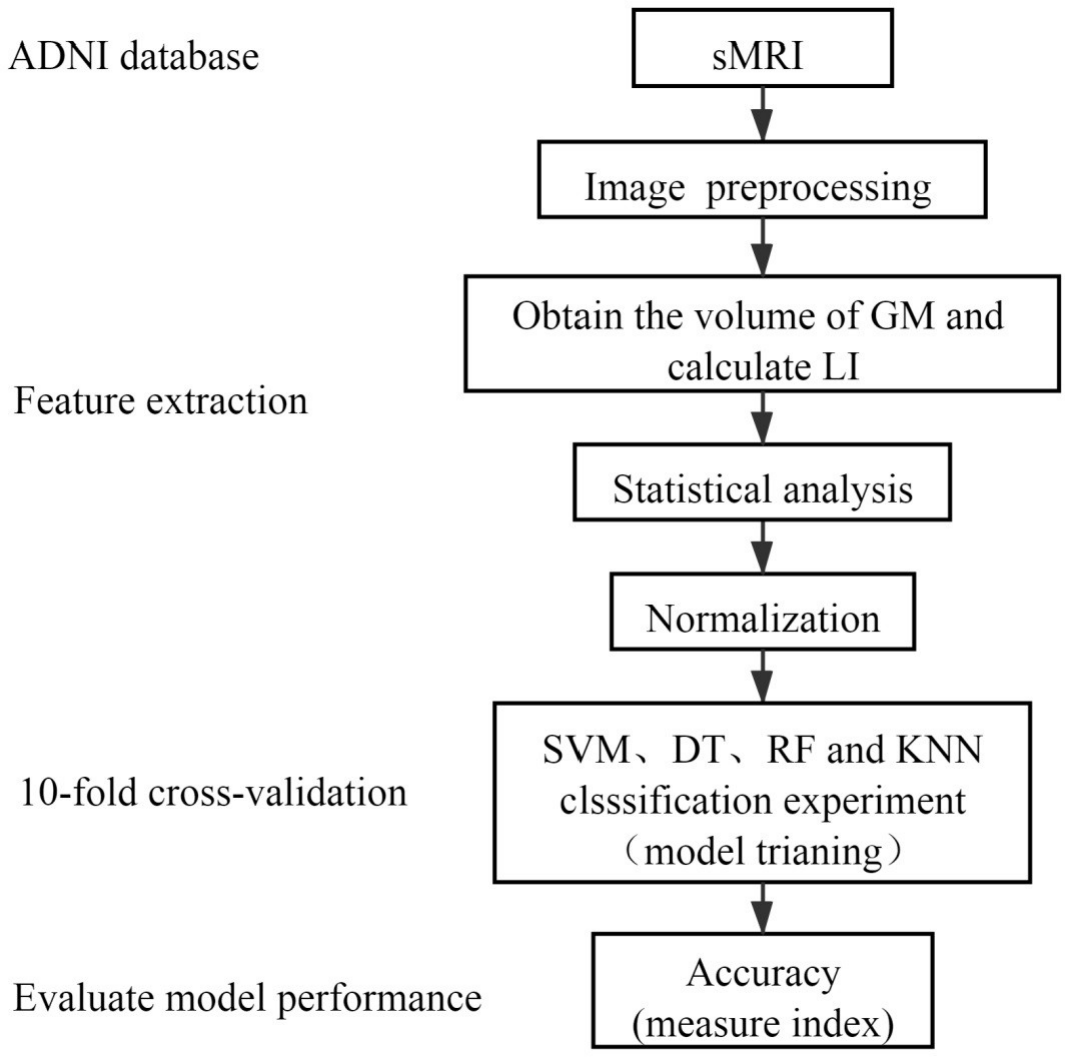
The classification algorithm flow chart for binary classification among NC, EMCI, LMCI and AD groups.

As indicated in the algorithm flow chart of Fig 1, the detailed classification algorithm for the considered four test groups are as follows. Just as stated in the above, the data samples containing 203 structural MRI images from 94 different subjects are firstly obtained from the ADNI dataset. Then, the MRI samples are pre-processed. After processing, calculate the GM volume on the AAL brain atlas, and then calculate the LI feature through the GM volume. Statistical analysis of ANOVA and the Kruskal-Wallis test is followed to extract classification feature. The obtained features of the GM volume in 64 different brain regions and LI for 15 different brain regions are normalized. Next, three classifier algorithms including RF, DT, KNN and SVM with RBF kernel, Linear kernel or Polynomial kernel are employed to realize binary classification among NC, EMCI, LMCI and AD groups. Using the 10-fold cross-validation method, the classification accuracy for each classifier algorithm is evaluated over 50 realizations of the result of 10-fold cross-validation. At last, the classification accuracies using the single feature of GM volume or LI are compared with that using two combined features of GM volume and LI.

In this work, the turning parameters for these employed classifier models are as follows. The turning parameters of SVM classifier with RBF kernel are optimized by grid search technique. The regularization parameter—C and the kernel coefficient—gamma are respectively optimized in the range of [2^Cmin, 2^Cmax] and [2^Gmin, 2^Gmax], in which the default values are Cmin = -8, Cmax = 8, Gmin = - 8 and Gmax = 8. Besides the SVM classifier with RBF kernel, the tuning parameters for other classifier algorithms are selected to optimize the classifier accuracy. For SVM classifier with polynomial kernel, the turning parameters of degree, regularization parameter—C and the kernel coefficient–gamma are chosen as 3, 1and 0.5, respectively. The tuning parameter of minleaf for DT classifier is 3. For RF classifier, the number of trees is 500, the max depth of each tree is default for the small sample size. The tuning parameter k for KNN classifier is 1.

### Classification performances

In this section, the classification performances are presented in detail. They include binary classification results across six test groups, comparison of classification results between different subject groups and different classification algorithms, and comparison with other works in the literature.

### Binary classification results

In this section, several classifier algorithms including RF, DT, KNN and SVM with RBF kernel, Linear kernel or Polynomial kernel are used to realize binary classification among NC, EMCI, LMCI and AD groups. The classification accuracies are outlined in Tables 1–6. The above two lines denotes the accuracies in the case that the single feature of GM volume in 64 different brain regions and the single feature of LI for 15 different brain regions are respectively considered, whereas the below line denotes the accuracies in the case that the newly introduced feature of LI is integrated into the original feature of GM volume.

**Table 1:** Classification results in NC versus EMCI under three kinds of features(%).

Classifier Feature	SVM			RF	DT	KNN
	RBF	Linear	Polynomial			
GM	88.8462	90.5128	93.6538	90.9179	75.0244	95.4269
LI	90.4487	67.4359	91.2821	90.1590	73.4821	90.4923
GM+LI	91.2821	92.8846	94.5513	91.3974	74.6538	95.4910

**Table 2:** Classification results in NC versus LMCI under three kinds of features(%).

Classifier Feature	SVM			RF	DT	KNN
	RBF	Linear	Polynomial			
GM	90.4545	92.3636	93.1818	90.1691	75.5800	93.8364
LI	93.2727	90.3636	93.2727	88.1273	77.0636	96.1564
GM+LI	96.0909	94.2727	94.0000	90.4145	77.1491	95.4291

**Table 3:** Classification results in EMCI versus LMCI under three kinds of features(%).

Classifier Feature	SVM			RF	DT	KNN
	RBF	Linear	Polynomial			
GM	91.6667	94.0000	94.7778	89.5311	76.0711	91.0000
LI	91.7778	85.3333	88.8889	86.8244	69.4800	91.2711
GM+LI	90.7778	91.8889	94.7778	90.6378	74.0289	92.4200

**Table 4:** Classification results in EMCI versus AD under three kinds of features(%).

Classifier Feature	SVM			RF	DT	KNN
	RBF	Linear	Polynomial			
GM	85.6667	87.6667	90.7778	89.2867	86.5644	92.8822
LI	91.0	77.6667	90.7778	89.0711	73.6178	90.7333
GM+LI	85.8889	92.8889	90.8889	91.1067	88.9733	94.2244

**Table 5:** Classification results in LMCI versus AD under three kinds of features(%).

Classifier Feature	SVM			RF	DT	KNN
	RBF	Linear	Polynomial			
GM	84.4643	88.5714	92.3214	89.5464	86.3321	91.9679
LI	89.8214	58.0357	82.1429	79.2321	71.1821	85.7821
GM+LI	88.2643	93.7500	92.3214	89.5964	87.3179	91.1464

**Table 6:** Classification results in AD versus NC under three kinds of features(%).

Classifier Feature	SVM			RF	DT	KNN
	RBF	Linear	Polynomial			
GM	95.1818	97.0909	94.3636	94.4582	90.9709	98.0800
LI	94.3636	86.7273	88.7273	92.7164	74.7873	93.0727
GM+LI	97.1818	98.0909	95.2727	94.6018	91.1018	98.2564

#### Classification results in NC versus EMCI

Table 1 shows the classification accuracies of NC group versus EMCI group under different classification algorithms. In the case of single feature of GM volume, the results indicate that the KNN classifier has the highest accuracy of 95.4269%. In the case of single feature LI, the results indicate that the SVM classifier with Polynomial kernel has the highest accuracy of 91.2821%. In the two cases, the lowest accuracies of 75.0244% and 73.4821% are all obtained by DT classifier. In the case of the combined feature of GM volume and LI, the highest accuracy and the lowest one are still achieved by KNN classifier and DT classifier, which are 95.491% and 74.6538%, respectively. What’s more, with the introduction of LI feature into GM volume feature, the classification accuracies under SVM classifiers with RBF kernel, Linear kernel or Polynomial kernel as well as RF classifier are improved significantly compared to those in the feature of GM, among which the accuracy under SVM classifier with Linear kernel has the most enhancement of 2.3718%, whereas the prediction accuracy under DT classifier is slightly decreased, with a decrease of 0.3706%.

#### Classification results in NC versus LMCI

The classification accuracies of NC group versus LMCI group under different classification models are displayed in Table 2. Under the circumstance of single feature of GM volume and single feature of LI, it turns out that the KNN classifier has the highest accuracy of 93.8364% and 96.1564% respectively, and the DT classifier has the lowest accuracy of 75.5800% and 77.0636% respectively. Under the circumstance of the combined feature of GM volume and LI, the DT classifier still has the lowest accuracy of 77.1491%, while the highest accuracy is achieved by SVM classifier with RBF kernel has the accuracy of 96.0909%. Furthermore, with the introduction of LI feature into the GM volume feature, the classification accuracies under RF classifier, DT classifier, KNN classifier as well as SVM classifiers with RBF kernel, Linear kernel or Polynomial kernel are all improved remarkably compared to those in the feature of GM. Especially the SVM classifier with RBF kernel has the most enhancement of 5.6364%.

#### Classification results in EMCI versus LMCI

Table 3 illustrates the classification accuracies of EMCI group versus LMCI group under three classification algorithms. Under the single feature of GM volume, the experimental results indicate that the SVM classifier with Polynomial kernel has the highest accuracy of 94.7778%, and the DT classifier has the lowest accuracy of 76.0711%. In the case of LI feature, KNN classifier has the highest classification accuracy of 91.2711% and DT has the lowest classification accuracy of 69.48%. Under the combined feature of GM volume and LI, the SVM classifier with Polynomial kernel and the DT classifier can still achieve the highest accuracy and the lowest one, which are 94.7778% and 74.0289%, respectively. With the addition of LI feature to GM volume, compared to those in the feature of GM the prediction accuracies of under SVM classifier with Polynomial kernel is unchanged, the prediction accuracies under RF classifier and KNN classifier are slightly improved, while the accuracies of DT classifier, SVM classifiers with Linear kernel and RBF kernel are decreased by 0.8889%, 2.1111% and 2.0422% respectively.

#### Classification results in EMCI versus AD

The classification accuracies of EMCI group versus AD group are depicted in Table 4 under different classification models. In the situation of single feature of GM volume, the results show that the KNN classifier has the highest accuracy of 92.8822%, and the SVM classifier with RBF kernel has the lowest accuracy of 85.6667%. In the case of LI feature, SVM classifier with RBF kernel has the highest classification accuracy and DT has the lowest classification accuracy, which are 91% and 73.6178%, respectively. In the situation of the combined feature of GM volume and LI, the SVM classifier with RBF kernel still has the lowest accuracy of 85.8889%, while the highest accuracy of 94.2244% is achieved by KNN classifier. As the LI feature is introduced into the GM volume feature, all the experimental accuracies of these classifiers are improved compared to those in the feature of GM, among which the most enhancement of 5.2222% is achieved by SVM classifier with Linear kernel.

#### Classification results in LMCI versus AD

The classification accuracies of LMCI group versus AD group are presented in Table 5 under different classifier algorithms. In the case of single feature of GM volume, the results suggest that the SVM classifier with Polynomial kernel has the highest accuracy of 92.3214%, and the SVM classifier with RBF kernel has the lowest accuracy of 84.4643%. Under the circumstance of single feature of LI, SVM with RBF kernel has the highest classification accuracy of 89.8214%, while SVM classifier with Linear kernel has the lowest accuracy of 58.0357%. With the introduction of LI feature into the GM volume feature, the highest accuracy is 93.7500% and the lowest one is 87.3179%, which are obtained by SVM with Linear kernel and DT classifier, respectively. The accuracies under RF classifier and SVM classifier with Polynomial kernel remain basically unchanged, the accuracy under KNN classifier is slightly decreased, however the classification accuracies under DT classifier, SVM classifier with RBF kernel or Linear kernel are improved significantly, which are 0.9858%, 3.8% and 5.1786%, respectively.

#### Classification results in AD versus NC

Table 6 depicts the classification accuracies of AD group versus NC group under different classifier algorithms. Under the single feature of GM volume, the results suggest that the KNN classifier has the highest accuracy of 98.08%, and DT classifier have the lowest accuracy of 90.9709%. Under the single feature of LI, the results show that the accuracy of SVM with RBF kernel has the highest accuracy of 94.3636%, while DT classifier have the lowest accuracy of 74.7873%. Moreover, when the LI feature is introduced into the GM volume feature, the highest accuracy and the lowest one are achieved by KNN classifier and DT classifier, which are 98.2564% and 91.1018% respectively. All the accuracies under different classification algorithms have been enhanced, with the most enhancement of 2% under SVM classifier with Linear kernel. In addition, when the LI feature is introduced into the GM volume feature, all the accuracies under five classification algorithms have been enhanced, with the most enhancement of 2% under SVM classifier with RBF kernel.

### Comparison of classification performances

In the following, firstly the classification performances between different subject groups are compared when fixing each classifier model. Then the classification performances between different classification algorithms are compared when fixing each binary subject groups. At last, the current classification performances are compared with the previous results in the literature.

#### Comparison between different subject groups

**Fig 2:**
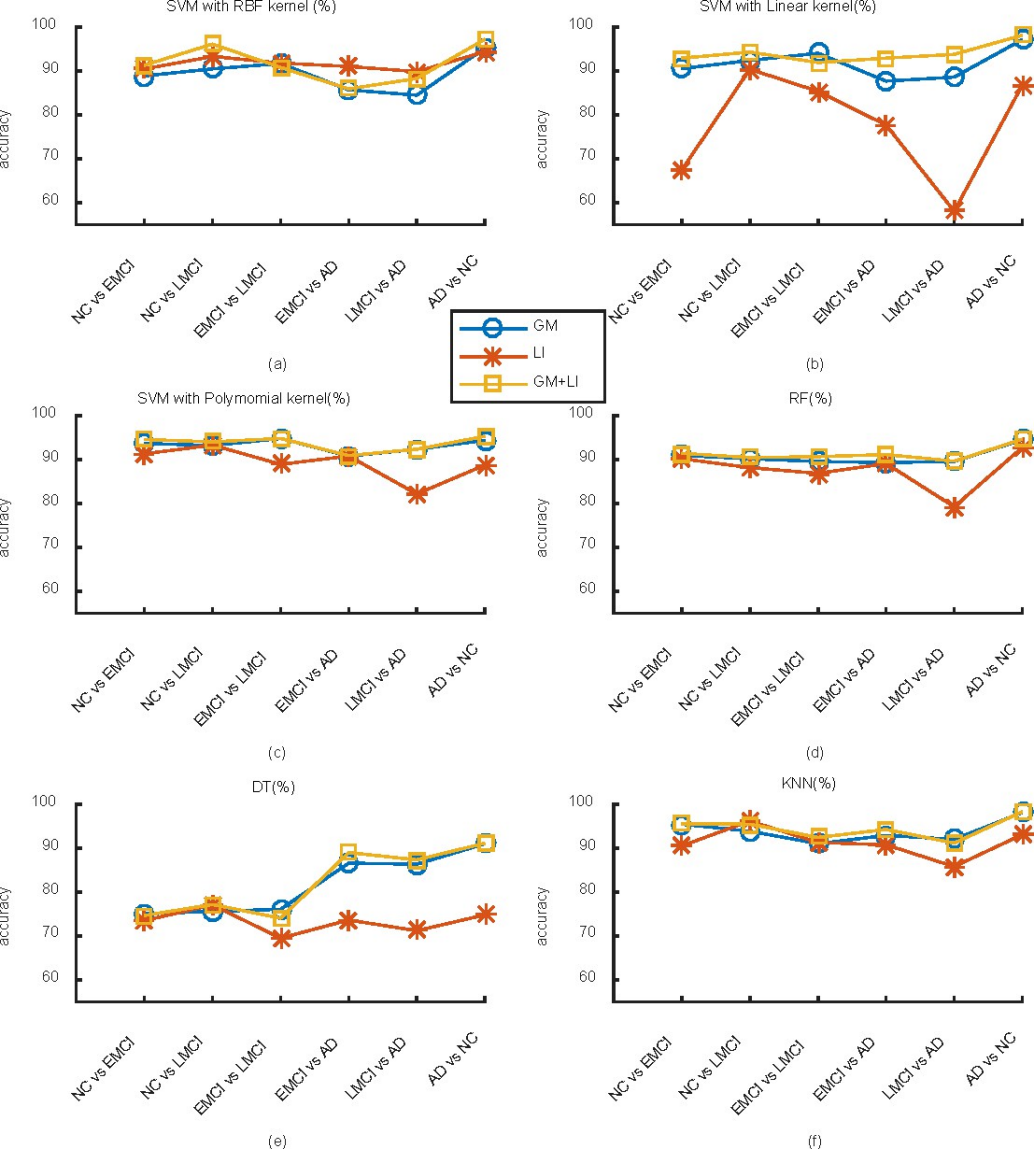
For each classification algorithm, the classification accuracies for binary subject groups NC versus EMCI, NC versus LEMC, EMCI versus LEMC, EMCI versus AD, LMCI versus AD, AD versus NC.

When fixing the SVM classifier with RBF kernel, linear kernel and polynomial kernel, RF classifier, DT classifier and KNN classifier, the classification accuracies for binary subject groups NC versus EMCI, NC versus LMCI, EMCI versus LMCI, EMCI versus AD, LMCI versus AD, AD versus NC are depicted in Fig 2A–2F, respectively. Comparing with these figures, some results can be obtained as follows. (1) For each classifier algorithm, the accuracies between the six subject groups are obviously different. Interestingly, the accuracies under the KNN classifier and the SVM classifier with polynomial kernel and with the RBF kernel are relatively stable among different groups, while the accuracies for different groups under SVM classifier with Liner kernel and DT classifier fluctuate severely. (2) For all the classification algorithms, the classification accuracy for AD versus NC group is almost the best, reaching the highest accuracy of 98.0909%. (3) By introducing the LI feature into the GM volume, the classification accuracies for most test groups are improved except for EMCI versus LMCI and NC versus EMCI groups. The highest improvement is 5.6364% under the SVM classifier model with RBF kernel. For NC versus EMCI group under DT classifier as well as EMCI versus LMCI group under SVM classifier with RBF kernel and linear kernel, the classification accuracies are decreased slightly, with the maximum decrease of 2.1111%.

#### Comparison between different classification algorithms

**Fig 3:**
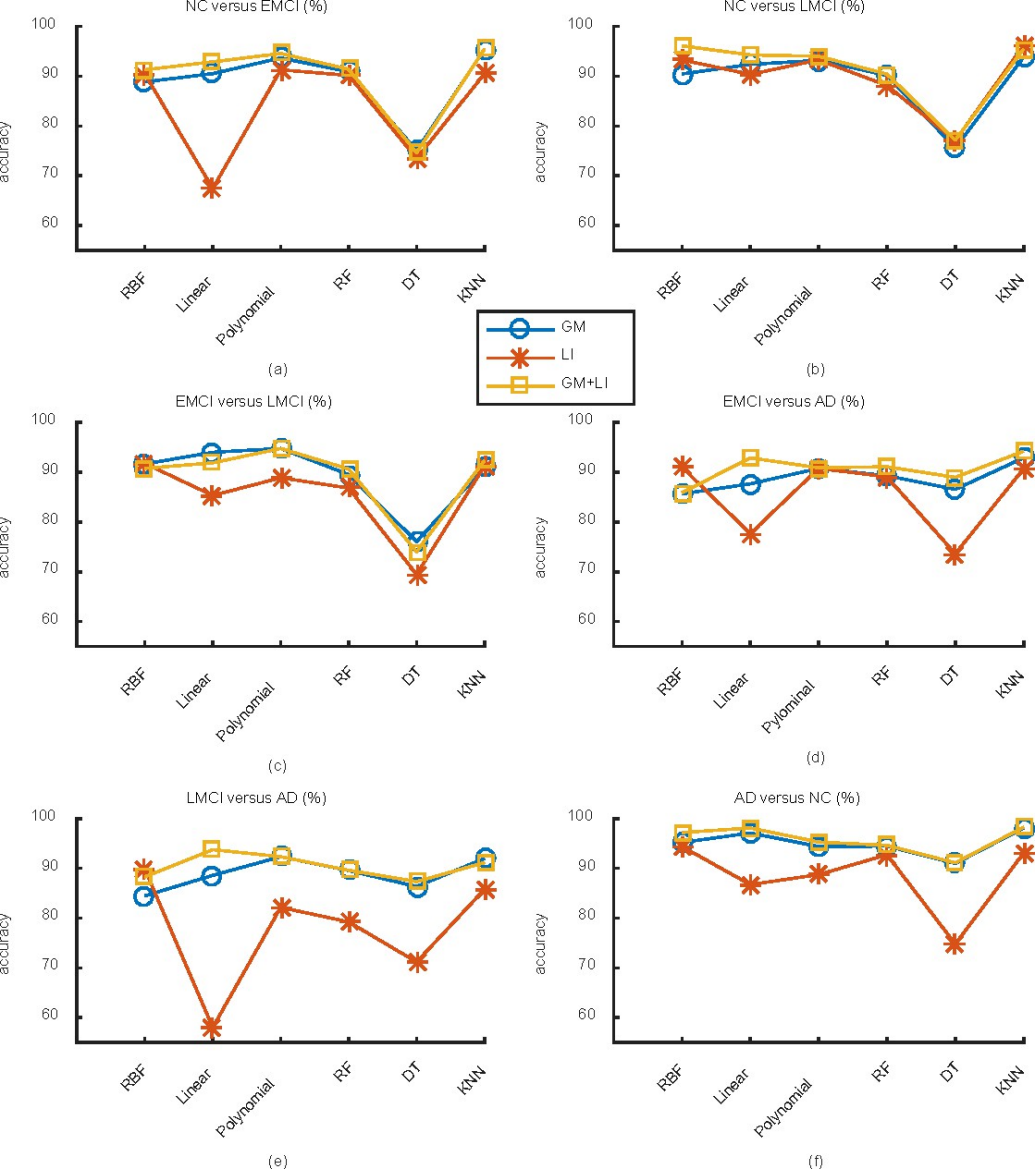
For each subject group, the classification accuracy under SVM classifier with RBF kernel, linear kernel and polynomial kernel, RF classifier, DT classifier and KNN classifier.

For each binary subject groups NC versus EMCI, NC versus LEMC, EMCI versus LMCI, EMCI versus AD, LMCI versus AD, AD versus NC, Fig 3A–3F illustrates the classification accuracies under SVM classifier with RBF kernel, linear kernel and polynomial kernel, RF classifier, DT classifier and KNN classifier, respectively. From these figures, some results can be summarized as follows. (1) For each subject groups, the accuracies under the different classification algorithms are distinctly diverse. Note that among the six binary subject groups, the accuracies for EMCI versus AD and NC versus AD groups are relatively stable and the classification accuracies for the rest subject groups fluctuate obviously, especially when the classification performance in the feature of LI is not included. (2) For all the six subject groups, the classification accuracies under the SVM classifier with polynomial kernel and the KNN classifier are relatively higher, while the accuracies under the DT classifier are mostly lowest. (3) By combining the LI feature with the GM volume feature, the classification accuracies for most test groups are enhanced, with the most enhancement 5.6364% for NC versus LMCI group under the SVM classifier model with RBF kernel. While for NC versus EMCI group under DT classifier as well as EMCI versus LMCI group under SVM classifier with RBF kernel and linear kernel, the classification accuracies are a little degraded, with the most reduction of 2.1111%.

### Comparison with other works

In the literature, there have been some results that identifying AD based on structural MRI. By extracting CSF and hippocampus volume on the AAL atlas, Ben et al. classified AD, MCI and NC group and obtained the accuracy of 87% for AD versus NC group [26]. On the basis of the volume of the hippocampus and entorhinal cortex, Fan et al. enhanced the classification accuracy of NC versus AD to 94.3% through a high-dimensional classification model [30]. Based on the T1-weighted MR scan GM segment, Klöppel et al. employed SVM classifier with Linear kernel to classify pathological AD patients and the elderly with normal cognition, where 96% of AD patients were classified correctly [31]. In our work, combing two features including GM volume and LI, the classification accuracy for AD versus NC is 98.2564% under the KNN classifier, which is also higher than the best classification accuracy of 92.4% for AD versus NC only using low-level feature from MRI [21].

## Conclusion and discussion

This work focuses on computer-aided diagnosis of AD and its prodromal stage including EMCI and LMCI. Employing the MRI images from ADNI dataset, the GM volume and LI based on AAL atlas are obtained. The classification feature containing GM volume in 64 different brain regions and LI for 15 different brain regions are firstly extracted by ANOVA and the Kruskal-Wallis test. Then, several classifier algorithms including RF, DT, KNN and SVM with RBF kernel, Linear kernel or Polynomial kernel are employed to realize binary classification among NC, EMCI, LMCI and AD groups. How the classification features and experimental algorithms influence the classification performances are explored. Using the 10-fold cross-validation method, the accuracies for binary classification are illustrated in Tables 1–6, Figs 2 and 3, from which some conclusions can be summarized as follows.

1. For different classifier models, the classification accuracy of NC group versus AD group is relatively stable especially in the feature of GM volume and the combined feature of LI and GM volume, which implies that the classification accuracy for AD versus NC is not dependent on these classifier algorithms. Moreover, no matter what classifier model is employed, the accuracy of AD versus NC is almost the highest across these binary classifications, with a best accuracy of 98.2564%. This indicates that the classification accuracies are affected by test groups, and a greater disease difference between groups is helpful to enhance classification accuracies.
2. For different subject groups, the SVM classifier algorithm with Polynomial kernel and the KNN classifier algorithm shows relatively stable and high classification accuracies, while DT classifier algorithm shows relatively unstable and lower classification accuracies. This implies that the classification accuracies are influenced by classifier model.
3. The classification performance in the feature of LI is poor compared with that in the case of GM feature i.e., the classification accuracies in the feature of LI are relatively low and unstable for most classifier models and subject groups. As the lateralization information is introduced into the original feature of GM volume, the classification accuracy for most binary subject groups is significantly improved, with a maximum increase of 5.6364%. However, the classification accuracies for some binary test groups are slightly decreased, including EMCI versus LMCI under DT classifier, SVM classifier with RBF kernel and Linear kernel, as well as NC versus EMCI under DT classifier, with a maximum decrease of 2.1111%. It can be seen that the feature of LI can effectively enhance the classification results for most test groups, which is helpful for early prediction of AD. When the disease difference between test groups is not striking (EMCI versus LMCI, NC versus EMCI), the prediction accuracy is slightly decreased by incorporating the LI. This may be due to the fact that when the boundary of the test groups is ambiguous, there is no distinct difference in brain lateralization, which makes the feature of LI can not play a positive role in classification prediction.

The above results indicate that various factors of subject samples, feature types and experimental algorithms influence classification performance remarkably, especially the newly introduced feature of LI is beneficial to improve the classification performances when the disease difference between test groups is distinct. As known that MCI patients are a high risk population for AD. Early diagnosis and intervention can greatly reduce the risk of AD. This work proposes a technique to classify AD progression based on comprehensive samples including NC, EMCI, LMCI and AD groups. Here MCI is divided into fine stages of early MCI and late MCI, compared to a general stage of MCI in most previous works. In addition, one may wonder how is the classification performance if the four groups of NC, EMCI, LMCI and AD are simultaneously classified. Using the combined feature of GM volume and LI, our further experimental results illustrate that the classification accuracies for the four groups under different classifier of SVM with RBF, Linear, Linear kernel, RF, DT and KNN are 84.8714%, 85.2648%, 88.109%, 84.2595%, 84.2595% and 71.3448%, respectively. The classification accuracies of four group classification are relatively lower than those obtained in binary classification. As known that four group prediction will be much more clinically important and more exciting, meanwhile, two group classification might be easier and more powerful. Thus, these obtained results are believed to shed new perspective on neuroimaging data analysis, which provides diverse theoretical basis for clinical recognition and diagnosis of AD in different situations of two subject groups or four subject groups.

At last, we want to point that besides the evaluation metric of classification accuracy there are some other metrics such as AUC, TPR and TNR to reflect the classification performance. Due to the various considered factors including three kinds of classification features, several kinds of classification algorithms and six groups of binary classification, this work only uses the evaluation metric of classification accuracy to study how classification features and experimental algorithms influence classification performance for simplicity. In addition, the data sample in this work consists of 203 structural MRI scans from 94 subjects. For the same subject, multiple images are collected at different time points. Although they may change over time, it is hard to control the dependence of different samples from the same subjects. Regretfully, multiple scans from the same subjects are treated as independent samples in this work. For the features selection, we extract the classification features using the 203 structural MRI scans by hypothesis testing methods (ANOVA and Kruskal-Wallis test). Then these 203 samples are split into training and validation sets by random divisions in the 10-fold cross-validation. Due to the randomness of sample selection, if one subject has multiple scans, some scans maybe divided into the training data and others maybe divided into the validation data. For the above two occasions there might be some shared information between training and testing dataset, which might lead to the over fitted classification results. These statements might be the limitation of the present results. In the future, we will increase the sample size and try to avoid the issue of overfitting by directly dividing different subjects into training and testing data with a ratio, performing variable selection and classification purely on training samples, and then test the results on the testing samples. On the same time, several evaluation metrics including AUC, TPR and TNR would be employed in combination to reflect the classification performance more thoroughly.
